# Long Noncoding RNA miR210HG as a Potential Biomarker for the Diagnosis of Glioma

**DOI:** 10.1371/journal.pone.0160451

**Published:** 2016-09-27

**Authors:** Weijie Min, Dongwei Dai, Jiaqi Wang, Dandan Zhang, Yuhui Zhang, Guosheng Han, Lei Zhang, Chao Chen, Xiulong Li, Yanan Li, Zhijian Yue

**Affiliations:** 1 Department of Neurosurgery, Changhai Hospital, Second Military Medical University, 168 Changhai Road, Shanghai, 200433, China; 2 Clinical Research Center, Changhai Hospital, Second Military Medical University, 168 Changhai Road, Shanghai, 200433, China; 3 Department of Neurosurgery, People’s Hospital of Yinan, Yinan, 276300, China; University of Navarra, SPAIN

## Abstract

**Background:**

Glioma remains a diagnostic challenge because of its variable clinical presentation and a lack of reliable screening tools. Long noncoding RNAs (lncRNAs) regulate gene function in a wide range of pathophysiological processes and are therefore emerging biomarkers for prostate cancer, hepatic cancer, and other tumor diseases. However, the effective use of lncRNAs as biomarkers for the diagnosis of glioma remains unproven.

**Methods:**

This study included 42 glioma patients and 10 healthy controls. lncRNA and mRNA microarray chips were used to identify dysregulated lncRNAs in tumor tissue and tumor-adjacent normal tissue, and SYBR Green–based miRNA quantitative real-time reverse transcription polymerase chain reactions were used to validate upregulated lncRNAs. A receiver operating characteristic curve analysis was conducted to evaluate the diagnostic accuracy of the lncRNA identified as the candidate biomarker.

**Results:**

miR210HG levels were significantly higher in tumor tissue than in tumor-adjacent normal tissue in participating glioma patients. Serum miR210HG levels were also significantly higher in glioma patients than in healthy controls. The receiver operating characteristic curve showed that serum miR210HG was a specific diagnostic predictor of acute pulmonary embolism with an area under the curve of 0.8323 (95% confidence interval, 0.7347 to 0.9299, *p* < 0.001).

**Conclusion:**

Our findings indicate that miR210HG could be an important biomarker for the diagnosis of glioma, and, as such, large-scale investigations are urgently needed to pave the way from basic research to clinical use.

## Introduction

Gliomas are the most aggressive and common type of primary adult brain tumor [1]. Anaplastic astrocytomas and glioblastoma multiformes (GBMs) are classified as high-grade gliomas (HGGs), which represent up to 50% of all primary brain gliomas and have the poorest prognosis [2]. The median survival length of patients with HGGs remains less than one year, even with aggressive surgery, radiation, and chemotherapy [3]. New prognostic indicators and effective therapeutic targets for gliomas are urgently needed.

Tools for the diagnosis of glioma range from analysis of genic mutations such as isocitrate dehydrogenase (IDH) mutation, chromosome deficiency assays, and the gold standards of diagnostic imaging, MRI and MRS [4–7]. Although extensively used as a screening tool, IDH mutation analysis is specific but not sensitive enough for glioma diagnosis [8]. New biomarkers with intensive diagnostic accuracy would greatly improve the diagnosis of glioma.

Long noncoding RNAs (lncRNAs) have recently been found to play important roles in numerous cellular functions [9], such as growth, reproduction, differentiation, and apoptosis [10–12]. LncRNAs are long, endogenous, single-stranded RNAs without coding functions. One function of lncRNAs is to downregulate gene expression by binding with messenger RNAs (mRNAs) and depressing mRNA translation or speeding mRNA degradation [13]. The expression patterns of numerous lncRNAs show multiform pathophysiologic appearances in different diseases [9]. We therefore have attempted to explore lncRNAs as sensitive and specific biomarkers for the diagnosis of glioma [14–18].

## Material and Methods

### Patient populations

Between February 2015 and July 2015, patients with glioma and healthy volunteers with negative IDH mutation assay tests were recruited from the Department of Neurosurgery at Changhai Hospital, Shanghai. According to existing guidelines, all glioma patients had undergone some combination of surgery, enhanced computed tomography (CT) brain scans, and immunohistochemistry assays on tumor tissue and/or tumor-adjacent normal tissue. Following glioma surgery, an analysis of chromosomal aberrations and karyotypes was performed for glioma patients to detect (or exclude) the glioma genotype combined with tissue from healthy volunteers in the absence of other tumor diseases. Pathological grading via an immunohistochemistry assay was then conducted according to glioma guidelines.

Briefly, high-grade (defined by the WHO as grades III and IV) gliomas are diagnosed through undifferentiated or anaplastic tumor tissue. During the study period, 28 glioma patients were confirmed to have high-grade gliomas, and these 28 patients were informed and enrolled in this study. Additionally, 10 healthy volunteers were enrolled as a control team. Of the 28 glioma patients, 10 patients were diagnosed with astrocytoma, 11 with glioblastoma, 4 with oligodendroglioma, and 3 with other types of glioma. Approval had previously been obtained from the ethical committees of the Second Military Medical University, and all participants involved gave written informed consent prior to commencement of the study.

### Blood samples

After informed consent, human peripheral blood samples were collected at Changhai Hospital in affiliation with Shanghai Military Medical University. The samples were left to stand in coagulant tubes for 30 minutes at 4°C, and serums were extracted after centrifuging. A total of 200 μl serum was used for miRNA quantitation assays using a quantitative real-time polymerase chain reaction (qRT-PCR) according to the kit manufacturer’s protocols. The remaining serums were stored at −80°C for further study [15,19].

### RNA isolation and quantitative reverse transcription PCR

The total RNA content of the tumor tissue and/or tumor-adjacent tissue samples was extracted using a TRIzol (Life Technologies, New York, USA) reagent according to the manufacturer’s protocols. Additionally, the total RNA of the serum samples was extracted using a TRIzol LS (Life Technologies) reagent, again according to the manufacturer’s protocols. Reverse transcriptions were performed using the Reverse Transcript Kit (Takara, Dalian, China), and qPCR was performed using a SYBR Green qPCR Kit (Takara) [20]. Amplification of glyceraldehyde-3-phosphate dehydrogenase (GAPDH) was performed in tissue samples as an endogenous control [21], and amplification of U6 was performed in serum samples as the same. Fold changes were calculated using the 2-ΔΔCt method. All reactions were performed in triplicate.

### LncRNA and mRNA microarray analysis

A lncRNA microarray assay (Agilent-045142 Human SBC LncRNA v4 4 × 180K) was performed by Biotechnology Corporation (Shanghai, China). LncRNA and mRNA microarray analysis was performed as described previously [22]. Briefly, the total RNA content was amplified and transcribed into fluorescent cRNA using a one-color Low Input Quick Amp Labeling Kit (Agilent Technologies, Santa Clara, USA), and the labeled cRNA was purified using an RNeasy Mini Kit (QIAGEN) and hybridized to the Agilent Human SBC LncRNA Microarray (4 × 180K, platform: GPL18180, Agilent Technologies).

Approximately 30,000 lncRNAs were collected from authoritative data sources, including NCBI_refseq, NCBI_other, Ensembl, UCSC, LNCRNA-DB, Agilent, ncRNA-SCAN, and published papers. Moreover, the correlations between approximately 31,000 mRNAs and lncRNAs were analyzed using this array. After 17 hours of hybridization, slides were washed in staining dishes using a Gene Expression Wash Buffer Kit (Agilent Technologies), and all slides were scanned on an Agilent Microarray Scanner with the following default settings: 1) channel: green, 2) scan resolution: 3 μm, 20 bit. All data were extracted using Feature Extraction Software 10.7 (Agilent Technologies), and dates were normalized using a quantile algorithm via Gene Spring Software 11.0 (Agilent Technologies).

### lncRNA target prediction

Most differentially expressed lncRNAs (fold change ≥ 5) in the microarray data were selected as potential targets [23], and two independent arithmetics were used. The first searched for target genes acting in cis. Using gene annotations from the UCSC Genome Browser (http://genome.ucsc.edu/), the lncRNA and mRNA sequences of potential target genes were visualized and paired. All genes transcribed within a 10 kb region downstream or upstream of lncRNAs were considered target genes. The second arithmetic was based on the sequence complementarity of mRNA with lncRNA and duplex energy prediction and assessed the impact of lncRNA binding with mRNA using BLAST software from the NCBI website (http://blast.ncbi.nlm.nih.gov/Blast.cgi) for the first round of screening. Finally, RNAplex software was used to choose trans-acting target genes [24].

### Statistical analysis

All data characterized were presented as means with standard deviations. The relative expression of lncRNA and mRNA was presented using the 2-ΔΔCt method [16,25], whereby the relative expression was calculated for the lncRNA and mRNA of interest (Ct gene of interest) relative to the endogenous control gene GAPDH (Ct internal control). The delta Ct for the tumor tissue group was compared with the delta Ct ± SD (where SD is the standard deviation of the average delta Ct of the group) for the normal tissue group or the healthy control group and tested with a one-way analysis of variance (ANOVA) for statistical significance.

If a significant difference was found, a T-test ANOVA was conducted when appropriate. After that, a receiver operating characteristic (ROC) curve analysis was performed with the lncRNA content of the tissue, distinguishing between tumor tissue and tumor-adjacent normal tissue. The area under the curve (AUC) was calculated to evaluate the diagnostic accuracy of the identified lncRNA. All analyses were performed using Microsoft Excel 2016, with statistically significant values of *p* < 0.05.

## Results

### Expression profiles of lncRNAs and mRNAs in the plasma of APE patients

**Fig 1:**
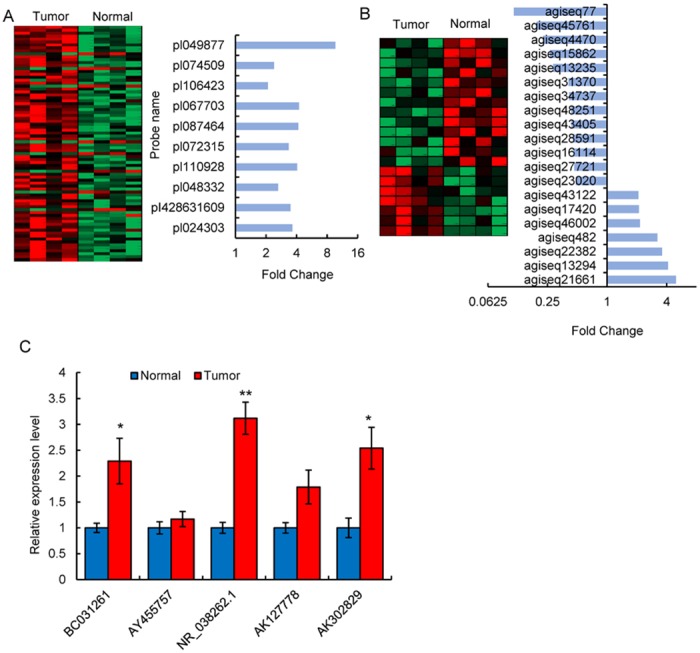
lncRNA and mRNA microarray chip tests in tumor tissue and tumor-adjacent normal tissue. (A) A lncRNA microarray chip test in 4 glioma patients. The left panel shows 80 differentially expressed lncRNAs, whereas the right panel shows the 10 most differentially expressed lncRNAs. (B) An mRNA microarray chip test in the same sample group. The left panel shows 20 differentially expressed mRNAs, and the right panel shows the fold change in mRNA levels. (C) A confirmatory qRT-PCR experiment on lncRNAs selected by a microarray chip and a BLAST test in the same sample group. ** *p* < 0.05.

The researchers identified 80 differentially expressed lncRNAs in tumor tissue as opposed to tumor-adjacent normal tissue (S1 Table). From these, 10 were found to be increased in tumor tissue (Fig 1A) and were selected for further analysis. In the same chip, 210 differentially expressed mRNAs were identified in tumor tissue as opposed to tumor-adjacent normal tissue (S2 Table), the sequences of 20 of which were found to have changed significantly in tumor tissue and were selected for further analysis (Fig 1B). Based on models of the complementary binding and degradation of nucleic acid chains, sequence comparisons were performed between selected lncRNA and mRNA using BLAST. BLAST scores were used to evaluate the complementary rate between lncRNA and mRNA (S1 Fig). As a result, four lncRNAs were thought to be responsible for a decrease in mRNA. After a confirmatory qRT-PCR experiment (Fig 1C), miR210HG was selected for further research based on observed changes (> 3.17fold), probability values (*p* < 0.05), and its complementary binding with BMP1.

### Validation of candidate miR210HG

**Fig 2:**
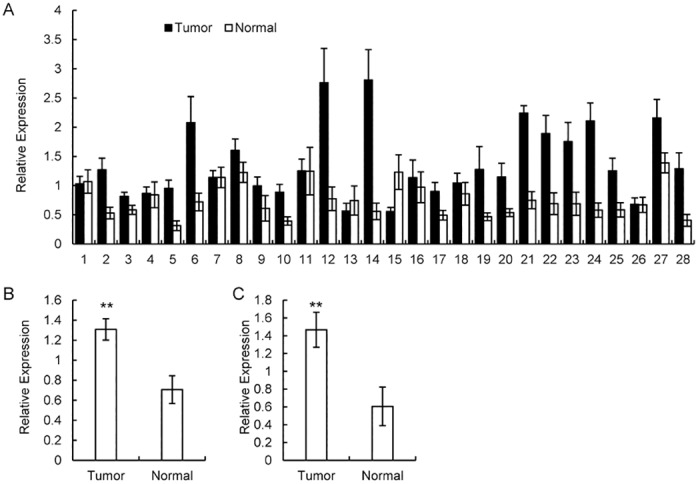
qRT-PCR tests on miR210HG in tumor tissue and tumor-adjacent normal tissue. (A) Relative expression in all 28 glioma patients. (B) qPCR comparison between tumor tissue and tumor-adjacent normal tissue. (C) qPCR comparison. qPCR data were normalized using GAPDH or U6. ** *p* < 0.05, * *p* < 0.01.

The relative contents of miR210HG were calculated using a SYBR Green qRT-PCR in all tissue samples from glioma patients (Fig 2A and 2B), the basic clinical characteristics of whom are shown in S3 Table. No significant differences in gender composition, IDH mutation, and chromosomal aberration were observed in the glioma patient groups (Table 1). The change in candidate miR210HG in tumor tissue versus tumor-adjacent normal tissue controls is shown in Fig 2B.

**Table 1:** Clinical characteristics of all tissue samples from glioma patients.

Group	p value
Gender	0.127424
Age	0.031573
WHO level	0.045516
Neuron	0.297623
CD99	0.051515
IDH mutation	0.193968
Syn	0.444534
Chromosome defective	0.289908

The raw data are transformed using the 2-ΔΔCt method and analyzed with one-way analysis of variance (ANOVA) methods. The p values were presented for statistical significance.

All these data were normalized by the contents of GAPDH, an endogenous reference mRNA widely used for its stable expression in tumor tissue. Moreover, the difference in miR210HG expression between tumor tissue and tumor-adjacent normal tissue controls was compared, and the same differences in miR210HG expression were obtained regardless of whether GAPDH or U6, an endogenous reference for lncRNA, was used as the normalizer (Fig 2C). This supports the assertion that GAPDH is a stable reference for lncRNA studies.

**Fig 3:**
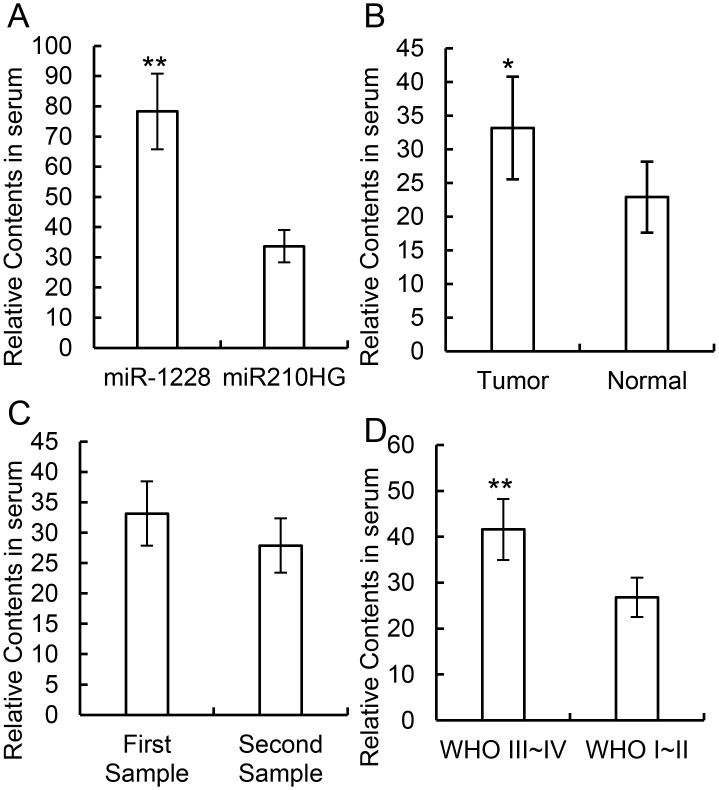
qRT-PCR tests on serum miR210HG in glioma patients and healthy controls. (A) Relative expression comparison between miR1228 and miR210HG. (B) Relative content of miR210HG in glioma patients and healthy controls. (C) Relative content of miR210HG in the first sample and the second sample (24 hours after the first sample). (D) Relative content of miR210HG comparison between high-risk (WHO III or IV) and low-risk glioma patients. ** *p* < 0.05, * *p* < 0.01.

To further study the diagnostic value of miR210HG, researchers collected the serum of 10 glioma patients and 10 healthy volunteers, and total RNA isolation along with a qRT-PCR was performed for miR210HG quantification. miR-1228 was also amplified as an endogenous control for its stable contents in the collected serum. At this stage, stable miR210HG could be detected in all serum samples (Fig 3A). The relative contents were then compared between the 10 glioma patients and the 10 healthy volunteers, with the result that plasma miR210HG levels were upregulated in the glioma patient group compared to the healthy volunteer controls (Fig 3B).

To confirm the repeatability of the assay, the relative expression levels of miR210HG in the serum gathered 24 hours after first sampling in three glioma patients was calculated, and no significant difference was found in miR210HG levels between these two groups, which indicates that this test is repeatable (Fig 3C). Serum miR210HG levels were also compared between high-risk (WHO III or IV) and low-risk (WHO I or II) glioma patients, and researchers found that miR210HG levels were relatively higher in the high-risk group (Fig 3D).

### ROC curve analysis of miR210HG for glioma

**Fig 4:**
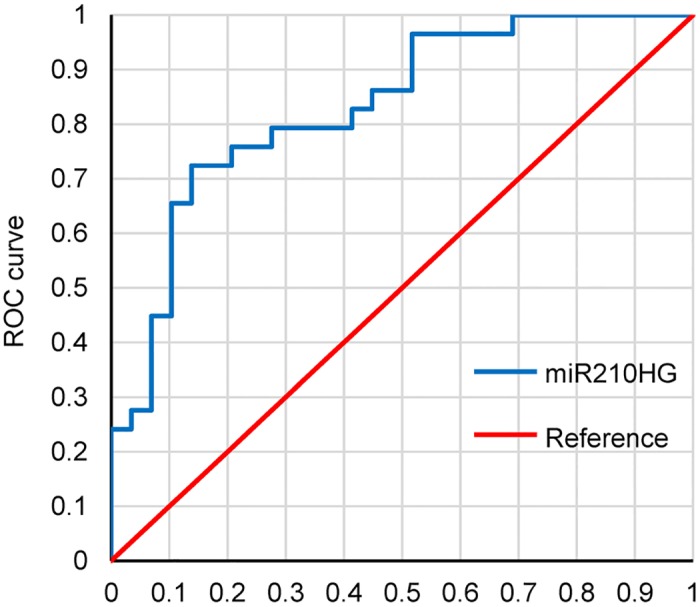
ROC curve tests on tumor tissue and tumor-adjacent normal tissue of glioma patients.

An ROC curve analysis was performed to analyze the diagnostic accuracy of plasma miR210HG. In a comparison between tumor tissue and tumor-adjacent normal tissue, the AUC was 0.8323 (0.7347 to 0.9279, 95% confidence interval, *p* < 0.001) (Fig 4). Using a Youden’s index of 0.5862 as the cutoff value for relative miR210HG content levels, the sensitivity and specificity of miR210HG as a biomarker for the diagnosis of glioma were 86.21% and 72.41%, respectively (Fig 4B).

## Discussion

Glioma has little specific presentation and is hard to diagnose. Although MRI and MRS have allowed for significant progress in the diagnosis of glioma, there remains an urgent need for a reliable biomarker and simple detection method for accurate diagnostic testing [26–37]. In this study, miR210HG has been identified as just such a potential biomarker for glioma.

An ideal biomarker facilitates test methods with high sensitivity and specificity, reproducible for the diagnosis of at least one disease [17,38]. lncRNAs are thought to be potentially suitable biomarkers for their high specificity and stability in tumors [17]. On the other hand, lncRNAs can be detected in human serum in a relatively stable form without degradation by RNase even under the complex environment in vivo, and specific lncRNA profiles in cancer patients have been reported [23]. Their relative stability and specificity in tumors thus make serum lncRNAs ideal biomarker candidates.

High sensitivity and specificity in detecting lncRNA via a qRT-PCR may create accurate cutoff concentrations for diagnosis. In recent years, no research has been undertaken on the profiles of serum lncRNAs in glioma patients. As demonstrated by miR210HG, identified in our research as a potential biomarker, the use of lncRNAs as minimally specify biomarkers for glioma could result in various valuable breakthroughs for the diagnosis of other diseases, as well.

MRI and MRS undergo wide clinical use [14,39–44]. Given the limitations of conventional MRI in distinguishing low-grade gliomas (LGGs) from HGGs, with an accuracy between 55% and 83%, researchers have explored advanced multiparametric magnetic resonance (MR) techniques including diffusion weighted imaging (DWI), diffusion tensor imaging (DTI), proton MR spectroscopy (MRS), and perfusion imaging [44]. On the other hand, in patients for whom MR is not possible, such as those with renal failure or metal implants, tests for miR210HG might prove useful as alternative diagnostic methods. In addition, miR210HG detection with custom diagnostic methods may improve screening efficiency.

The presence of serum miR210HG could distinguish tumor tissue from tumor-adjacent normal tissue with an AUC of 0.8323, and the data indicates that miR210HG could be used as a potential biomarker for glioma diagnosis. In this study, miR210HG levels in the tumor tissue of 28 glioma patients were 3.5 times higher than in tumor-adjacent normal tissue, but the miR210HG levels in the serum of 10 glioma patients were only two times higher than in the serum of healthy volunteers. This disparity may be due to the content difference of lncRNA levels in serum versus in tumor tissues used in our research data.

Our study has a number of limitations regarding lncRNA research. First, the number of glioma patients involved in this experiment was relatively small, and independent large studies of glioma patients are needed to verify the results obtained from this small group. Second, the relationship between lncRNA miR210HG and mRNA BMP1 in glioma patients must be established further with research. It would be useful to identify whether combining the miR210HG content and values of CD99 could enhance the sensitivity and specificity of glioma diagnosis. Some samples in this research did not reveal the presence of CD99, as all the collected serum was used for RNA isolation. Further research is needed to resolve the source of the tissue. The CD99 test in glioma patients has a high sensitivity without enough specificity because CD99 is also present in patients with other cancers, such as gastric cancer. In this research, the specificity of miR210HG in glioma diagnosis was better than that offered by the CD99 test.

Third, further research is required to evaluate the additive benefits of miR210HG for glioma diagnosis when used with other diagnostic methods, such as immunohistochemistry tests. Fourth, the biological function of miR210HG is unclear. Circulating lncRNA is thought to play a role in maintaining the microenvironment in vivo, and it has been speculated that serum miR210HG can have some pathogenic effects in glioma patients. Finally, the pathobiological mechanism of miR210HG expression and its relationship with BMP1 in glioma patients is unclear. Prior research has demonstrated that the secretion of lncRNA by exosomes or apoptotic bodies may cause the upregulation of lncRNA.

## Supporting Information

S1 Fig The Blast Score between lncRNA and mRNA. (JPG)

S1 Table 80 differentially expressed lncRNAs in tumor tissue as opposed to tumor-adjacent normal tissue. (XLSX)

S2 Table 8 most differentially expressed mRNAs in tumor tissue as opposed to tumor-adjacent normal tissue. (XLSX)

S3 Table The basic clinical characteristics of all samples. (XLSX)
