# Supplementary figures and images for: Long Noncoding RNA miR210HG as a Potential Biomarker for the Diagnosis of Glioma
Source: PLoS One. 2016 Sep 27;11(9):e0160451. doi: 10.1371/journal.pone.0160451 (PMC5038942; doi:10.1371/journal.pone.0160451)

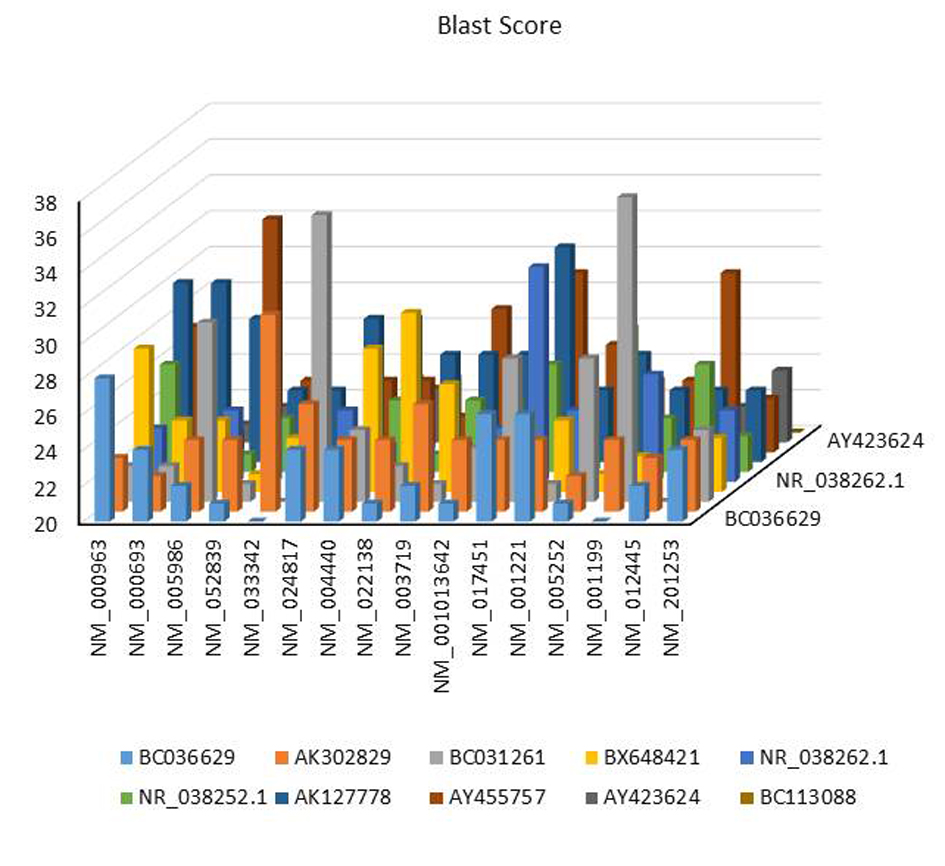

Supplement: S1 Fig — (JPG) [file pone.0160451.s001.jpg]
